# Turner syndrome: language profile of young girls at 12 and 24 months of age

**DOI:** 10.1186/s11689-021-09401-1

**Published:** 2021-11-04

**Authors:** Debra B. Reinhartsen, Emil Cornea, Margaret DeRamus, Angelia B. Waitt, Rebecca Edmondson Pretzel, Rebecca C. Knickmeyer, Marsha L. Davenport, John H. Gilmore, Stephen R. Hooper

**Affiliations:** 1 Carolina Institute for Developmental Disabilities, University of North Carolina-Chapel Hill, Chapel Hill, NC USA; 2 Department of Psychiatry, School of Medicine, University of North Carolina at Chapel Hill, Chapel Hill, NC USA; 3 Orange County Schools, Hillsborough, NC USA; 4 Department of Psychiatry & Carolina Institute for Developmental Disabilities, School of Medicine, University of North Carolina-Chapel Hill, Chapel Hill, NC USA; 5 Department of Pediatrics, School of Medicine, University of North Carolina-Chapel Hill, Chapel Hill, NC USA; 6 Department of Allied Health Sciences, School of Medicine, University of North Carolina-Chapel Hill, Chapel Hill, NC USA

**Keywords:** Receptive language, Expressive language, Turner syndrome, Infants, Toddlers, Neurodevelopmental disorders, Symbolic language, Social language

## Abstract

**Background:**

Turner syndrome (TS) is a genetic disorder associated with complete or partial absence of an X chromosome affecting approximately 1/2000 live female births. Available evidence suggests that, in the school-age years, girls with TS often require speech and language services; however, little is known about the language development of infants and toddlers.

**Method:**

This study (*N* = 31) explored the language profiles of 12- and 24-month-old girls with TS, as well as the percentage of girls who might be “at risk” for language delays. We also followed a subset of 12-month-old girls with TS to 24 months of age to determine the stability of the 12-month findings.

**Results:**

Although all mean scores were within the average range at both time points, results revealed a higher prevalence of 24-month-old girls with TS “at risk” for receptive language difficulties. In addition, expressive language skills significantly exceeded receptive language skills at both time points. We found 12-month-old girls to be “at risk” for social and symbolic difficulties based on clinical assessment; only symbolic difficulties were significant based on caregiver report. At 24 months, clinical assessment indicated greater use of speech sounds and words than normative expectations. Caregivers reported greater use of speech sounds, and also, greater use of gestures. Although some changes occurred over a 1-year time span (12 to 24 months), all mean test scores remained within the average range and the changes in the percentage of girls manifesting “at risk” status on either the *PLS-4* or *CSBS-DP* were non-significant.

**Conclusions:**

Although within normal limits, receptive language skills were found to be significantly lower than expressive language skills at both ages. Social and symbolic communication skills also were in the average range, with both showing significant improvement from 12 to 24 months based on clinical assessment. Caregiver report found that use of gestures and production of speech sounds not only improved from 12 to 24 months, but also exceeded normative expectations. Findings suggest the presence of relatively intact speech and language abilities during the first 2 years of life, with perhaps some emergent concerns for receptive language development. Ongoing developmental surveillance will be important.

## Background

Turner syndrome (TS) is a genetic disorder associated with complete or partial absence of an X chromosome affecting approximately 1/2000 live female births. Diagnosis can be made prenatally through the detection of sex chromosome abnormalities; however, many times the diagnosis is missed or delayed [1]. Researchers in a single center study conducted in the UK [2] found that the mean age of diagnosis was 5.89 (± 5.3) years, ranging from prenatal to 17.9 years. Ten percent were diagnosed antenatally, 16% in infancy, 45% in childhood (1 to 12 years), and 20% in adolescence (12 to 18 years). A larger Dutch study found similar results [3]. Growth hormone and estrogen replacement therapies that encourage skeletal growth and pubertal-related changes are deemed primary treatments, so delayed diagnosis can be problematic with respect to the initiation of such treatments. Although wide variability exists, common phenotypic features of girls with TS include short stature, abnormalities in craniofacial development, recurrent middle ear infections, hearing loss, cardiovascular, and renal abnormalities, lymphedema, delayed or absent pubertal development, and learning disabilities [4]. School-age girls with TS typically have been reported to demonstrate average intellectual functioning; however, nonverbal abilities tend to be significantly lower than verbal abilities, and weaknesses tend to occur in the areas of visual-spatial skills, executive functioning, and pragmatic language skills [5–7]. Additionally, individuals with TS have been described as experiencing difficulties with oral reading fluency and executive language functions, such as strategic retrieval during oral narratives [8–13]. Early detection of these features allows for more timely interventions and, therefore, the potential for improved quality of life.

### Typical infant and toddler language development

Around 12 months of age, infants with typical development, transitioning into toddlerhood, move out of the prelinguistic stage and begin to produce meaningful single words (e.g., “mama,” “dada”) in the presence of the referent for a specific communicative function or intent (e.g., to request, refuse, or gain attention). Prior to the development of words, symbolic gestures, such as pushing away, reaching, or waving, are used to express communicative functions; therefore, gestures continue to be used and often are paired with vocalizations at this age. Infants at this age also follow simple commands, especially when accompanied with visual or symbolic gestural cues [14, 15]. Additionally, there are a number of pre-linguistic predictors that precede later language development and include the symbolic abilities to understand words, use objects, and use gestures. These symbolic abilities generally occur prior to the use of words and have been found to be sensitive indicators of subsequent communication and social interactions [16–18]. For infants and toddlers with TS, the integrity of these pre-linguistic functions and related communication capabilities remains unknown.

### Language and communication function in girls with Turner syndrome

A study conducted by Temple and Shephard [19] found that some language skills appear to be superior in TS during childhood but equivalent to the general population as adults. They reported that younger girls (ages 4 to 5 years) with TS exhibited significantly higher receptive and expressive language and phonological skills than peers who are typically developing (TD), but when young adults with TS (ages 19 to 23 years) were compared to their TD peers, this language and phonological advantage was no longer evident. Furthermore, when compared to those who are TD, girls with TS, ages 8 to 12 years, have been shown to demonstrate exceptional vocabulary knowledge [20] and superior reading abilities [21].

Despite reports of language strengths in this population, communication disorders and speech difficulties in girls with TS also have been described. Van Borsel et al. [22] conducted a survey of communication problems in 128 individuals with TS, ranging in age from 2.4 to 58.8 years of age. Survey results indicated that 24%, at some point, had received speech and language services, 15% of which were for language delays and about 14% with articulation-related concerns. This rate is higher than the 8% rate seen in the general population as reported by the 2012 National Health Interview Survey [23]. Van Borsel et al. suggested that oral anomalies, in addition to chronic ear infections and hearing loss in children (often requiring surgical placement of tympanostomy tubes into the eardrum to promote ventilation and drainage of fluid in the middle ear), could adversely affect speech production. Craniofacial abnormalities also can be present and include a small and retrognathic jaw, a narrow palate with prominent ridges, malocclusion, and low oral muscle tone.

The pragmatic language skills in girls with TS also have been examined. Pragmatic language skills allow for the effective social use of language through verbal (i.e., turn taking, talking differently to different audiences, providing background information to unfamiliar listeners) and nonverbal means related to symbolic understanding and use (i.e., use and recognition of gestures, facial expressions, eye contact, body language). Using the Social Responsiveness Scale (SRS) as one measure, Hong et al. [5] examined selected aspects of social competence in girls with TS (42 pre-estrogen treatment; 32 TD), who ranged in age from 3 to 12 years. Results indicated that when compared to TD peers, girls with TS had a mild-moderate decrease in Social Cognition which measures an individual’s capacity to interpret social cues, both verbally and nonverbally (e.g., understands the real meaning of a conversation, has a sense of humor, understands the meaning of others’ tone of voice and facial expressions, is imaginative/good at pretending). Taken together, these difficulties are significant for their impact on general communication but also other functions, such as social engagement and peer interaction.

### Current study

Despite the available literature and documented language differences, there is no research on emergent language functioning and language development in girls with TS during the infant and toddler period. This is a significant developmental period when speech and language functions are beginning to manifest and also a developmental period where early intervention could be initiated.

To address this gap in the literature, we examined the core and social language features of 12- and 24-month-old girls with TS and compared them to available normative data in order to determine the level and pattern of language abilities. We first hypothesized that girls with TS, at 12 and 24 months of age, would show a mean language score within the average range, but that they would manifest selected difficulties in symbolic and social language, perhaps secondary to nonverbal and pragmatic language weaknesses documented in the literature for preschool and school-age girls with TS [5–7]. Second, based on research suggesting that a higher number of girls with TS require speech and language services during the school-age years, we examined this question in our sample. We hypothesized that, at 12 and 24 months of age, the percentage of girls with TS in the “at risk” range (i.e., one or more standard deviations (SD) below the mean) would be significantly greater than the percentage of TD children in the “at risk” range (i.e., 16% expected under the assumption that the scores are normally distributed).

Furthermore, we conducted an exploratory analysis to examine changes in language abilities from 12 to 24 months of age using the subset of 12-month-old infants with TS who had a follow-up evaluation at 24 months. These analyses examined changes in score performance and in the “at risk” classification over the 1 year period.

## Methods

### Participants

The sample comprised a total of 31 infants who were diagnosed with TS either prenatally (as an incidental finding or due to observed abnormalities on ultrasound) or shortly after birth by karyotype (due to dysmorphic features, lymphedema, or heart problems). They were recruited nationally by advertisement on the internet and through the Turner Syndrome Society of the USA, as well as through the TS Clinic at a large southeastern university. Twenty-six infants were enrolled and assessed at approximately 12 months of age. Of these, 17 returned for a second assessment at approximately 24 months of age. Of the 17, two were missing Behavior Sample data and one was missing Caregiver Questionnaire data at the 24-month time point. An additional five infants were enrolled and assessed only at 24 months. Ninety percent of the participants were Caucasian, 3% were African American, and 6% were of mixed race. All but two parents had at least a high school education, with approximately 39% having a 4-year college degree. Thirteen of the infants with TS had heart abnormalities, two of which were surgically corrected. Thirteen infants underwent at least one surgery in the first 2 years of life (primarily for tympanostomy tubes). Pertaining to developmental skills, two infants had an Early Learning Composite Standard Score (*ELCSS*, mean = 100; SD = 15) on the Mullen Scales of Early Learning (MSEL) [24] below 70, i.e., very low, during at least one study visit. One had a higher score at an earlier visit and the other received a higher score at a later visit, while the remaining participants had *ELCSS* scores in the low average to average range.

### Measures

The Preschool Language Scale, Fourth Edition (PLS-4) [25] is a measure used to assess receptive and expressive language skills in infants and young children ages birth through 6 years, 11 months. Reliability and validity of the *PLS-4* are strong. The normative standards for receptive and expressive language as well as total language are a mean of 100 and a SD of 15 with an average range of 86–114. The “at risk” population is defined as having scores 85 or below. Data were obtained through elicitation of tasks, observation, and parent report.

The Communication and Symbolic Behavior Scales, Developmental Profile (CSBS-DP) [26] is a norm-referenced, standardized measure of seven pre-language skills (emotion and eye gaze, communication, gestures, sounds, words, understanding, and object use) as well as three composite scores. Each scale and composite score have a normative mean of 10, SD of 3, and average range of 8–12. The “at risk” population is defined as having scores < 7. The *CSBS-DP* also yields a total standard score that has a mean of 100, SD of 15, and average range of 86–114; with “at risk” individuals scoring 85 or below. Data are collected via the Behavior Sample, which is completed by the clinician while the child interacts with the parent and examiner in the room, and the Caregiver Questionnaire completed by a parent/caregiver. The CSBS-DP was designed to use a combination of face-to-face assessment procedures with young children and caregiver report and maintains good psychometric properties.

The participants were assessed using these measures. The *PLS-4* was administered, and the CSBS-DP Behavior Sample obtained at 12 and 24 months of age. The CSBS-DP Caregiver Questionnaire also was completed at both time points.

### Data analyses

In the absence of an appropriate comparison group of TD peers, we addressed the primary research question by performing one-sample *t* tests to determine whether the participants’ mean PLS-4, CSBS-DP Behavior Sample, and CSBS-DP Caregiver Questionnaire scores were significantly different from the population normative mean at 12 and 24 months.

To further address the lack of an actual TD group, we performed a simulation study with 2000 randomly generated comparison samples using the population normative mean and standard deviation, tested the mean scores of each such comparison sample with the participants’ mean scores, and computed the empirical *p* values. The comparison samples were generated to reflect the available overall cognitive functioning, socioeconomic status (SES), and history of tympanostomy tube insertion (for chronic ear infections) characteristics of the standardization sample for each language measure. We considered the ELCSS, maternal education, and history of tympanostomy tube insertion (yes/no) as proxies for those characteristics, respectively. For the general population, we used the information available in the literature: ELCSS (mean = 100; SD = 15) and tympanostomy tube insertion rate of per 100 per year in the 2010–2011 US general child population [27]. According to the CSBS-DP manual, maternal education for the standardization sample of the CSBS-DP Behavior Sample has a mean of 15.2 years with a SD of 2.3 and for the Caregiver Questionnaire, a mean of 15.0 years with a SD of 2.2. No such information for the PLS-4 standardization sample was available to us. Therefore, we compared the performance of the TS group with each simulated TD group using ANCOVA with two samples, controlling for ELCSS, maternal education, and tympanostomy tube insertion for the CSBS-DP Behavior Sample and CSBS-DP Caregiver Questionnaire only. Because the results from the simulated study were similar to those of the one-sample *t* test, we chose to present the results from the one-sample *t* test analyses, which were obtained for all three measures. Furthermore, for the CSBS-DP measures, we highlighted the nuances of the findings from the two different methods.

To address our second research question, we tested whether the percentage of girls with TS who were in the “at risk” range was equal to the expected 16%, under the assumption that the participants’ scores were normally distributed. As our sample is small (*n* < 30) for each measure (PLS-4, CSBS-DP Behavior Sample, and CSBS-DP Caregiver Questionnaire), we performed exact chi-square tests to make inferences about the equality of the percentage of our “at risk” TS girls to the expected 16% for each subtest and composite score at ages 12 and 24 months separately.

For our exploratory analysis, we used paired *t* tests to investigate whether the performance score of the girls with TS significantly changed from 12 to 24 months in each outcome across the three measures. We also performed McNemar’s exact tests [28, 29] across the outcomes of the three measures to assess whether the “at risk” status classification significantly changed from 12 to 24 months.

All statistical hypothesis tests were two-tailed and conducted at a significance level of .05. The false-discovery rate (FDR) correction [30] for multiple comparisons was applied for all statistical tests within each measure (PLS-4, CSBS-DP Behavior Scale, and CSBS-DP Caregiver Questionnaire) separately, at both the 12 and 24 months age points, as well as for the change in measure over the 1-year period. All statistical analyses were performed using SAS statistical software, version 9.4.

## Results

### Comparison to core and social language normative data at 12 months of age

#### Preschool Language Scale-4

As seen in Table 1, the infant girls with TS who were tested at age 12 months (*n* = 26) scored in the average range in the area of core language as measured by the mean Total Language Score. Their mean AC and EC scores also were within the average range, but the mean AC score was lower, and the mean EC was higher than the corresponding normative means. One-sample *t* tests showed that neither the Total Language Score nor the AC or EC scale scores at 12 months of age were significantly different from the *PLS-4* normative data.

**Table 1:** Means and standard deviations of standard scores and percentages of “at-risk” infants and toddlers with TS on PLS-4, CSBS-DP Behavioral Sample, and CSBS-DP Caregiver Questionnaire

Variable	12 months					24 months		
	Mean scores ***t*** test ***(H***_***0***_***: μ***_***TS*pop**_ ***=μ***_***0***_***)***		At-risk exact ***χ***^***2***^***-***test ***(H***_***0***_***: %***_***TS*pop**_ ***= 16)***		Mean scores ***t*** test ***(H***_***0***_***: μ***_***TS*pop**_ ***= μ***_***0***_***)***		At-risk exact ***χ***^***2***^***-***test ***(H***_***0***_***: %***_***TS*pop**_ ***= 16)***	
	M ± SD	***p***	***%*** (***n***)	***p***	M ± SD	***p***	***%*** (***n***)	***p***
***PLS-4*** (*n*_12_ = 26, *n*_24_= 22)								
Auditory comprehension	96.5 **±** 11.7	.139	23.1 (6)	.418	93.0 **±** 11.4	.009*	31.8 (7)	.072
Expressive communication	105.0 **±** 12.7	.055	3.8 (1)	.109	99.9 **±** 11.5	.971	9.1 (2)	.420
**Total language**	100.8 **±** 12.6	.746	11.5 (3)	.611	96.3 **±** 12.0	.166	22.7 (5)	.562
***CSBS-DP BS*** (*n*_12_ = 25, *n*_24_ = 19)								
*Social*								
Emotion and eye gaze	9.1 **±** 1.7	.010*	4.0 (1)	.166	12.1 **±** 3.9	**.033**	15.8 (3)	> .999
Communication	8.6 **±** 2.8	.025*	48.0 (12)	< .001*	11.1 **±** 4.3	.279	31.6 (6)	.106
Gestures	8.2 **±** 3.1	.010*	36.0 (9)	.012*	10.1 **±** 3.6	.950	31.6 (6)	.106
*Speech*								
Sounds	9.6 **±** 2.0	.322	24.0 (6)	.413	11.6 **±** 2.4	.008*	5.3 (1)	.238
Words	10.4 **±** 1.5	.149	0.0 (0)	**.049**	12.5 **±** 3.4	.005*	5.3 (1)	.238
*Symbolic*								
Understanding	9.5 **±** 1.9	.178	4.0 (1)	.166	10.4 **±** 3.7	.672	15.8 (3)	> .999
Object use	7.7 **±** 2.9	< .001*	60.0 (15)	< .001*	11.1 **±** 3.3	.182	15.8 (3)	> .999
Composite								
Social	8.7 **±** 2.8	.031*	36.0 (9)	.012*	11.1 **±** 3.9	.256	21.1 (4)	.756
Speech	9.7 **±** 1.9	.464	12.0 (3)	.787	12.2 **±** 2.5	.001*	5.3 (1)	.238
Symbolic	8.0 **±** 2.7	.001*	52.0 (13)	< .001*	10.3 **±** 3.0	.703	21.1 (4)	.756
**Total standard score**	90.2 **±** 12.1	< .001*	36.0 (9)	.012*	107.6 **±** 17.6	.075	10.5 (2)	.570
***CSBS-DP CQ*** (*n*_12_ = 26, *n*_24_ = 20)								
*Social*								
Emotion and eye gaze	10.3 **±** 3.2	.676	34.6 (9)	.016*	11.9 **±** 3.2	**.015**	10.0 (2)	.564
Communication	10.2 **±** 3.1	.799	15.4 (4)	> .999	11.8 **±** 3.9	.052	10.0 (2)	.564
Gestures	9.9 **±** 3.4	.863	23.1 (6)	.418	14.4 **±** 4.8	< .001*	20.0 (4)	.759
*Speech*								
Sounds	9.5 **±** 2.6	.341	23.1 (6)	.418	13.2 **±** 4.0	.002*	5.0 (1)	.234
Words	10.0 **±** 2.6	.939	23.1 (6)	.418	11.4 **±** 3.1	.069	5.0 (1)	.234
*Symbolic*								
Understanding	8.5 **±** 3.1	**.024**	38.5 (10)	.005*	11.7 **±** 5.1	.151	25.0 (5)	.353
Object use	9.0 **±** 3.0	.107	34.6 (9)	.016*	9.1 **±** 2.3	.081	20.0 (4)	.759
Composite								
Social	9.9 **±** 2.9	.789	30.8 (8)	.056	11.4 **±** 2.5	**.021**	5.0 (1)	.234
Speech	9.8 **±** 2.7	.777	23.1 (6)	.418	11.4 **±** 2.9	**.044**	5.0 (1)	.234
Symbolic	8.8 **±** 2.9	**.046**	42.3 (11)	.001*	9.1 **±** 2.3	.095	30.0 (6)	.188
**Total standard score**	94.9 **±** 14.3	.082	30.8 (8)	.056	101.4 **±** 12.4	.619	15.0 (3)	> .999

*Note*. ***μ***_***TS*pop**_ ***=*** mean in the TS population, ***μ***_***0***_ = normative mean. Boldfaced *p* < .05, *p after FDR correction < .05

In addition, when examining the data, we noted that EC scores were generally higher than AC scores in 12-month-old girls with TS. This was reflected by an 8.50-point discrepancy in the mean scores. We ran a paired *t* test and found that this difference was statistically significant, *t*(25) = 4.85, *p* < .001 (*n* = 26). Given that nearly one-half of our participants had surgery for tympanostomy tube insertion in the first 2 years of life, and that language performance may be affected by overall development and socioeconomic status, we also conducted an ANCOVA of the score differences (i.e., EC minus AC), by tympanostomy tube insertion (yes/no) adjusting for ELCSS and maternal education. None of the covariates had a statistically significant effect on the score differences. A *t* test showed that the least square (LS) mean of the score differences remained statistically significant (LS mean of difference = 8.63, *t*(21) = 4.29, *p* < .001, *n* = 25). Thus, there is evidence in our data to support the idea that girls with TS performed significantly higher in expressive language than receptive at the age of 12 months.

#### Communication and Symbolic Behavior Scales-Developmental Profile

Similarly, for the CSBS-DP Behavior Sample (*n* = 25), the mean total standard score of infant participants was within the average range, as were all mean composite scores (Table 1). The Social and Symbolic Composites were at the lower end of the average range and lower than the normative means. The infants with TS also showed mean scores in the average range for all seven language scales. The two scales measuring symbolic language, symbolic understanding and object use, were in the average range, but object use was at the lower end of this range. When the scores from the TS group were statistically compared to the normative expectations for the CSBS-DP Behavior Sample, one-sample *t* tests followed by FDR correction showed the TS infants were rated significantly lower, not only on the total standard score, *t*(24) = − 4.07, *p* < .001, but also on the social composite, *t*(24) = − 2.29, *p* = .031, and symbolic composite, *t*(24) = − 3.59, *p* = .001. In addition, four individual scales, social emotion and eye gaze, *t*(24) = − 2.78, *p* = .010; social communication, *t*(24) = − 2.39, *p* = .025; social gestures, *t*(24) = − 2.81, *p* = .010; and symbolic object use_,_ *t*(24) = − 3.90, *p* = < .001, were found to be significantly lower than the normative expectations (Table 1).

Results of the CSBS-DP Caregiver Questionnaire (*n* = 26) mirrored those of the CSBS-DP Behavior Sample (Table 1). The mean total standard score, social, speech, and symbolic composite mean scores, and all seven individual scale mean scores of the infants with TS were within the average range. The symbolic composite and its related symbolic understanding scale mean scores were somewhat lower than normative expectations. The lower performances in the two outcomes were the only ones found to be statistically significant when one-sample *t* tests were performed. However, unlike with the CSBS-DP Behavior Sample, when the scores from the TS group were statistically compared to the normative expectations from the CSBS-DP Caregiver Questionnaire, no results remained significant after the FDR correction.

The simulation study’s ANCOVA analyses, controlling for ELCSS, maternal education, and tympanostomy tube insertion, yielded as significant, the same results as those from one-sample *t* tests for both CSBS-DP measures. However, the significance of lower performances by the infants with TS, when compared with the simulated TDs in the social composite and the three individual social scales of the CSBS-DP Behavior Sample, faded after FDR, showing just a trend of significance (.05 ≤ *p* < .10).

### Percentage scoring one or more standard deviations below the mean at 12 months of age

#### Preschool Language Scale-4

Although the mean scores for the infants with TS were in the average range on the PLS-4, there was wide ranging variability in the scores achieved at age 12 months. Approximately 23% of the 26 infants were in the “at risk” range for AC (Table 1), while only 3.8% and 11.5% were “at-risk” for EC and Total Language, respectively. However, exact chi-square tests showed no significant differences from the normal curve expectation of 16%.

#### Communication and Symbolic Behavior Scales-Developmental Profile

The CSBS-DP Behavior Sample results indicated that of the 25 participants, 12%, 36%, 52%, and 36% scored in the “at risk” range in speech, social, and symbolic composites and total standard score, respectively (Table 1). Exact chi-square tests, followed by FDR correction, showed that percentages were significantly higher than normal curve expectations of 16% only in social, *χ*^2^(1) = 7.44, *p* = .012, and symbolic, *χ*^2^(1) = 24.11, *p* < .001, composites, and in the total standard score, *χ*^2^(1) = 7.44, *p* = .012. For the individual scales of the CSBS-DP Behavior Sample, the percentages of being at “at risk” ranged from 0% (speech words) to 60% (symbolic object use), the latter being nearly a four-fold increase in what would be expected. Statistically, the percentage of girls with TS who were in the “at risk” range was significantly higher than 16% in social communication, *χ*^2^(1) = 19.05, *p* < .001; social gestures, *χ*^2^(1) = 7.44, *p* = .012; and symbolic object use, *χ*^2^(1) = 36.01, *p* < .001, after the FDR correction.

The *CSBS-DP* Caregiver Questionnaire results indicated that of the 26 infant participants, 23%, 31%, and 42% scored in the “at risk” range in speech, social, and symbolic composites, respectively (Table 1). Exact chi-square tests showed that the percentage was significantly higher than normal curve expectations only for the symbolic composite score, *χ*^2^(1) = 13.39, *p* = .001. For the individual scales of the CSBS-DP Caregiver Questionnaire, percentages ranged from 15% (social communication), which would be in line with normal curve expectations, to 38% (symbolic understanding), which represents approximately a 2.4-fold increase. The percentage of girls with TS who were in the “at risk” range was significantly higher than the expected 16% in social emotion and eye gaze, *χ*^2^(1) = 6.70, *p* = .016; symbolic understanding, *χ*^2^(1) = 9.76, *p* = .005; and symbolic object use, *χ*^2^(1) = 6.70, *p* = .016. All significant results survived the FDR correction.

### Comparison to core and social language normative data at 24 months of age

#### Preschool Language Scale-4

As seen in Table 1, the mean total standard score for the 24-month-old girls with TS (*n* = 22) was within the average range. Their mean AC and EC scores also were within the average range, but the AC mean was lower than the normative mean. As with the 12-month-old infants with TS, one-sample *t* tests showed that the 24-month-old toddlers’ EC and Total Language scores were not significantly different from the PLS-4 normative expectations. However, the AC mean of the toddlers with TS was found to be significantly lower than the PLS-4 normative expectations and remained significant after FDR correction, *t*(21) = − 2.87, *p* = .009.

As at age 12 months, we noted that most EC scores were higher than AC scores in 24-month-old girls with TS. This was reflected by a 6.86-point discrepancy in the mean scores. We ran a paired *t* test and found that this difference was statistically significant, *t*(21) = 5.04, *p* < .001 (*n* = 22). We also ran an ANCOVA of the score differences (i.e., EC minus AC), by tympanostomy tube insertion adjusting for ELCSS and maternal education. None of the covariates had a statistically significant effect on the score differences. A *t* test showed that the LS mean of the score differences remained significant (LS mean of differences = 6.99, *t*(16)=4.95, *p* < .001, *n* = 20). Thus, our data provide evidence to support the idea that girls with TS performed significantly higher in expressive language than receptive language at both 12 and 24 months of age.

#### Communication and Symbolic Behavior Scales-Developmental Profile

For the CSBS-DP Behavior Sample, the mean total standard score for the 24-month-old girls with TS (*n* = 19) was within the average range, as were social, speech, and symbolic composite and all seven individual scale mean scores (Table 1). When the scores from the TS group were statistically compared to normative expectations, one-sample *t* tests, followed by FDR correction, showed that toddlers with TS were rated significantly higher in the speech composite, *t*(18) = 3.83, *p* = .001, as well as in the individual scales of speech sounds, *t*(18) = 3.01, *p* = .008, and speech words, *t*(18) = 3.18, *p* = .005. Although the social emotion and eye gaze scale showed higher scores than the normative standards, the result did not remain significant after the FDR correction.

Results of the CSBS-DP Caregiver Questionnaire at 24 months (*n* = 20) generally mirrored those of the CSBS-DP Behavior Sample (Table 1). The mean total standard score and social, speech, and symbolic composite mean scores were within the average range. The girls with TS also obtained mean scores in the average range for all seven language scales. When the scores from the toddler TS group were statistically compared to normative expectations, one-sample *t* tests followed by FDR correction showed that toddlers with TS were rated significantly higher in social gestures, *t*(19) = 4.09, *p* = < .001, and speech sounds, *t*(19) = 3.50, *p* = .002. For girls with TS, *t* tests also showed significantly higher performance than the normative means on the social emotion and eye gaze scale, as well as social and speech composites, but the significance did not survive the FDR correction.

The simulation study’s ANCOVA analyses, controlling for ELCSS, maternal education, and tympanostomy tube insertion, yielded statistically significant results consistent with those from the one-sample *t* test analysis. In addition, the higher performances by toddlers with TS in social composite and social communication were significant for both CSBS-DP measures. It also was found that toddlers with TS performed significantly higher in the total standard score of the CSBS-DP Behavior Sample. All these results for the CSBS-DP Behavior Sample remained significant after FDR. Out of the significant results for the CSBS-DP Caregiver Questionnaire, all remained significant after FDR except those in social and speech composites, which faded to the trend of significance level (.05 ≤ *p* < .10), consistent with the *t* test analyses.

### Percentage scoring one or more standard deviations below the mean at 24 months of age

#### Preschool Language Scale-4

At the 24-month assessment, there was notable variability in the range of scores achieved on the PLS-4 by the girls with TS. Approximately 32% of the 22 toddlers were in the “at risk” range for AC (Table 1**)**. Although this percentage appeared somewhat high, the exact chi-square test did not find it significantly higher than the normal curve expectations of 16%. The EC (9%) and total language (23%) percentages also were not found to be significantly different from normal curve expectations.

#### Communication and Symbolic Behavior Scales-Developmental Profile

The CSBS-DP Behavior Sample results indicated that of the 19 toddler participants, 5% in speech composite and 21% in symbolic and social composites, scored in the “at risk” range (Table 1). For the individual scales of the CSBS-DP Behavior Sample, these percentages ranged from 5% in both speech sounds and speech words to 32% in both social communication and social gestures. Exact chi-square tests showed that none of these percentages were significantly different from the normal curve expectation of 16%.

Similarly, the CSBS-DP Caregiver Questionnaire results indicated that of the 20 toddler participants, 5% in speech and social composites and 30% in symbolic composite scored in the “at risk” range (Table 1). For the individual scales of the CSBS-DP Caregiver Questionnaire, the percentages ranged from 5% in both speech sounds and speech words to 25% in symbolic understanding. Exact chi-square tests showed that none of these percentages were significantly different from the normal curve expectation of 16%.

### Changes in standard scores from 12 to 24 months of age

For this exploratory examination of changes from 12 to 24 months, we considered the participants who were assessed at 12 months and also had a follow-up at 24 months. There were 17 such participants on the PLS-4, 15 on the CSBS-DP Behavior Sample, and 16 on the CSBS-DP Caregiver Questionnaire.

The AC, EC, and total language scores on the PLS-4 showed slightly downward trends but remained relatively stable over the 1-year period (Table 2). Paired *t* tests showed that none of these decreases were statistically significant.

**Table 2:** Assessment of changes in standard scores and “at-risk” classifications from 12 and 24 months for infants with TS who have 24-month follow-up on PLS-4, CSBS-DP Behavioral Sample, and CSBS-DP Caregiver Questionnaire

Variable	Mean scores 12 to 24 months Paired ***t*** test			“At-risk” 12 to 24 months McNemar’s exact test		
	12-months M± SD	24-months M ± SD	***p***	12 months % (***n***)	24 months % (***n***)	***p***
***PLS-4*** (*n* = 17)						
Auditory comprehension	98.2 + 12.2	94.2 + 11.3	.257	23.5 (4)	29.4 (5)	> .999
Expressive communication	106.4 + 13.8	101.8 + 10.4	.187	5.9 (1)	5.9 (1)	> .999
**Total language**	102.6 + 13.6	98.0 + 11.3	.188	11.8 (2)	17.6 (3)	> .999
***CSBS-DP BS*** (*n* = 15)						
*Social*						
Emotion and eye gaze	9.1 + 1.9	11.8 + 3.8	.016*	6.7 (1)	20.0 (3)	.625
Communication	8.7 + 3.0	10.9 + 4.0	.061	46.7 (7)	26.7 (4)	.375
Gestures	8.5 + 2.9	9.8 + 3.6	.303	26.7 (4)	33.3 (5)	> .999
*Speech*						
Sounds	10.1 + 1.9	11.9 + 2.2	.057	20.0 (3)	0.0 (0)	.250
Words	10.7 + 1.8	12.8 + 3.0	.018*	0.0 (0)	0.0 (0)	> .999
*Symbolic*						
Understanding	10.0 + 2.0	10.4 + 3.7	.624	0.0 (0)	13.3 (2)	.500
Object use	8.4 + 3.2	10.9 + 2.6	.010*	53.3 (8)	13.3 (2)	**.031**
***Composite***						
Social	9.1 + 2.9	10.8 + 3.7	.138	26.7 (4)	20.0 (3)	> .999
Speech	10.2 + 1.9	12.5 + 2.2	.015*	6.7 (1)	0.0 (0)	> .999
Symbolic	8.9 + 2.8	10.3 + 2.7	.098	46.7 (7)	20.0 (3)	.219
**Total standard score**	93.2 + 13.4	108.3 + 16.9	.008*	26.7 (4)	6.7 (1)	.250
***CSBS-DP CQ*** (*n* = 16)						
*Social*						
Emotion and eye gaze	10.8 + 3.1	11.6 + 3.4	.455	25.0 (4)	12.5 (2)	.500
Communication	10.0 + 3.1	11.8 + 4.1	.061	18.8 (3)	12.5 (2)	> .999
Gestures	9.9 + 3.4	14.4 + 4.8	.002*	18.8 (3)	18.8 (3)	> .999
*Speech*						
Sounds	9.8 + 3.0	14.1 + 4.0	< .001*	18.8 (3)	6.3 (1)	.500
Words	9.9 + 2.5	11.4 + 3.0	.110	25.0 (4)	6.3 (1)	.375
*Symbolic*						
Understanding	8.8 + 3.7	11.6 + 5.1	**.022**	31.3 (5)	25.0 (4)	> .999
Object use	8.7 + 2.9	9.3 + 2.2	.333	37.5 (6)	18.8 (3)	.375
***Composite***						
Social	9.9 + 3.0	11.1 + 2.5	.053	31.3 (5)	6.3 (1)	.125
Speech	9.9 + 2.8	11.8 + 3.0	**.045**	18.8 (3)	6.3 (1)	.500
Symbolic	8.9 + 3.1	9.3 + 2.3	.481	37.5 (6)	25.0 (4)	.500
**Total standard score**	95.1 + 14.7	102.1 + 12.8	**.017**	31.3 (5)	18.8 (3)	.500

*Note*. Boldfaced *p* < .05, * *p* after FDR correction < .05

For the CSBS-DP Behavior Sample (Table 2), there was a positive shift in the Total Standard score as well as all composite and individual scale scores from 12 to 24 months of age. Paired *t* tests followed by FDR correction showed increases with statistical significance in the total standard score, *t*(14) = 3.12, *p* = .008; speech composite, *t*(14) = 2.77, *p* = .015; social emotion and eye gaze, *t*(14) = 2.75, *p* = .016; speech words, *t*(14) = 2.67, *p* = .018; and symbolic object use, *t*(14) = 3.00, *p* = .010.

For the CSBS-DP Caregiver Questionnaire, there also was a positive shift for all scores (Table 2). Although positive in nature, all changes in scores were deemed to be within normal variation. Paired *t* tests showed that the increases in total standard score, speech composite, social gestures, speech sounds, and symbolic understanding were statistically significant; however, only the significance of increases in social gestures, *t*(15) = 3.84, *p* = .002, and speech sounds, *t*(15) = 4.09, *p* < .001, survived FDR correction.

### Changes in “at risk” classification from 12 to 24 months of age

On the PLS-4, the percentages of toddlers with TS manifesting “at risk” classification were similar at both time points on AC and EC as well as total language (Table 2). McNemar’s exact tests showed that the “at risk” status did not significantly change from 12 to 24 months of age on any of the three PLS-4 scales.

For the CSBS-DP Behavior Sample, there also were some notable changes in the percentages of participants deemed “at risk” over the 1-year time period. Clinician ratings showed a decline in the percentage of “at risk” individuals in each of the composite scores as well as the total standard score, particularly in the symbolic composite and total standard score. For the individual scales, the percentage of “at risk” participants decreased on social communication, speech sounds, and symbolic object use and increased on social emotion and eye gaze, social gestures, and symbolic understanding. Of note, none of the 15 girls with TS at the 1 year follow-up were in the “at risk” range for speech words at either time point. McNemar’s exact tests found that the change in the “at risk” status from 12 to 24 months was significant only in symbolic object use, i.e., a significant percentage of participants improved their performance over the 1 year period and were no longer considered to be “at risk” at 24 months. However, the result did not remain significant after FDR correction. For the CSBS-DP Caregiver Questionnaire, all composite and total standard scores showed a decrease in the percentage of “at risk” participants at 24 months of age. For individual scales, all of them also showed a decrease in the percentage of “at risk” participants except for social gestures, which showed no change. McNemar’s exact tests showed that in none of the outcome measures were the changes in the “at risk” status from 12 to 24 months statistically significant.

## Discussion

In order to determine the level and pattern of language abilities in 12- and 24-month-old girls with TS, we examined core and social language features using the PLS-4, CSBS-DP Behavior Sample, and CSBS-DP Caregiver Questionnaire. The purpose of our study was three-fold, to determine whether (1) there were symbolic and social language difficulties despite average receptive and expressive language scores, (2) the percentage of girls with TS in the “at risk” category would be significantly greater than the percentage of their TD peers, and (3) there were notable changes in language abilities from 12 to 24 months. First, our findings indicated that girls with TS, indeed, had average receptive and expressive language scores; however, girls with TS performed statistically significantly higher in expressive language than receptive language at both time points, and at 24months receptive language was lower than normative expectations. In addition, while significant social and symbolic difficulties were found in girls with TS who were 12 months of age, this was not the case for the 24-month-old girls with TS. Second, in neither group of 12- nor 24-month-old girls with TS, was the percentage of those at risk found to be significantly higher than the normal curve expectation in receptive or expressive language. However, a significantly higher percentage of the 12-month-old girls with TS were in the “at risk” range for social and symbolic language when compared to TD peers. Comparatively, there were no significant findings of 24-month-old girls with TS in the “at risk” category when compared to normative expectations. Finally, when examining AC, EC, and total language skills over the 1-year period (from 12 to 24 months of age), the changes in standard scores and those who were in the “at risk” category remained stable. On the CSBS-DP Behavior Sample, a statistically significant increase in mean scores over the 1-year period was noted in the areas of emotion and eye gaze, words, and object use, while on the CSBS-DP Caregiver Questionnaire, significant increases were noted in gestures and sounds mean scores. The “at risk” status within the group of girls with TS did not significantly change over the 1-year period for either measure.

### Infants and toddlers demonstrate average core and social language abilities with relative difficulties in symbolic and social language

We first hypothesized that girls with TS would show a mean language score within the average range but that there would be selected difficulties associated with social and symbolic communication based on pragmatic weaknesses (verbal and nonverbal) reported in the literature in preschool [5] and school-age girls [5–7]. With respect to core language abilities, our preliminary findings revealed relatively intact language skills across both receptive and expressive domains with all scores being within the average range. We suspected this would be the case, given the pattern of language abilities described for older girls with TS; however, we did not anticipate two subsequent findings: (1) that the 24-month-old toddlers’ receptive language mean would be significantly lower than normative expectations, and (2) at both time points, 12 and 24 months of age, mean expressive language scores would significantly exceed mean receptive language scores.

A higher expressive than receptive language pattern has been found in other populations, such as individuals with autism spectrum disorder (ASD) [31, 32]; however, there are potential explanations for why the finding in girls with TS might be different from that found in children with ASD. Individuals with ASD may produce delayed echolalia or scripted phrases within the correct context; however, if not in their own vocabulary repertoire, they may not understand each word [33, 34] or phrase they say, nor have the ability to use each word flexibly. Studies conducted by McDaniel et al. [33] and Woynaraski et al. [34] found that children with ASD presented with a smaller size discrepancy in receptive-expressive vocabulary than their peers with typically developing language who had the same level of vocabulary. In addition, the participants with ASD comprehended fewer words than was anticipated relative to their spoken words which contributed to an atypical language profile. Given that individuals with ASD are capable of producing longer learned utterances, they tend to have the expressive language skills to obtain credit for many items on the EC scale of the PLS-4. Many of the early items on the EC scale can be credited through observation and/or caregiver report (e.g., babbles with inflection, says five words, combines sounds in consonant-vowel-consonant or vowel-consonant-vowel combinations), whereas more of the AC scale items must be elicited (e.g., following directions; identifying objects, pictures, body parts, items of clothing). For the girls with TS (and similar to those with ASD [33]), expressive language scores may have been elevated because they often were observed to be inattentive or not motivated to show what they knew and refused to engage with elicited/on demand tasks. Based on findings from a recent study, this behavior may not be unusual in infants with TS [35]. Pretzel et al. [35] indicated, based on caregiver report, there were a larger number of TS infants with low persistence to task completion, negative mood, and poor management of their behavior when approaching or withdrawing from various situations.

Because pragmatic language delays have been documented in preschool- and school-age girls with TS, we also expected our sample might show selected difficulties in social and symbolic language which are indicators of subsequent communication and social interaction abilities [16–18]. We indeed did find this to be true at the 12-month time point; however, these relative difficulties were no longer evident at 24 months. While social and symbolic communication concerns disappeared over the 12- to 24-month time period, we found speech sounds and speech words emerged at 24 months as a strength, which may not be surprising, given that some studies have reported significantly higher phonological skills [19] and vocabulary knowledge [20] in girls with TS than in girls who are TD during childhood. Based on the CSBS-DP Caregiver Questionnaire, none of the mean scores were significantly different than the normative data at 12 months. At 24 months of age, however, not only did speech flourish, but there was an increase, above normative expectations, in the use of gestures. Although only speculative, perhaps by 24 months, by becoming more familiar with their child’s speech, speech patterns, and gestures, parents became more accurate in describing what their child could do. Alternatively, perhaps parents became more biased reporters due to overstatement or misinterpretation of their child’s communicative intents. Even though the CSBS-DP Behavior Sample mean scores in the areas of social emotion and eye gaze, social communication, social gestures, and symbolic object use became nonsignificant when compared to the normative data at 24 months of age, the findings of being significantly lower at 12 months of age alert us to the need to monitor social and symbolic language skills in infants with TS due to its potential impact on later pragmatic language abilities.

### Percentage of infants and toddlers with TS at-risk for language problems

While some studies have reported superior language skills in young girls with TS [19], others [22] have reported that a high percentage of girls had received speech and language services, 15% of which were for language. For this reason, we suspected there would be a higher prevalence of infant and toddler girls with TS “at risk” for core and social language difficulties. The percentage of infant and toddler girls with TS “at risk” for receptive language difficulties was not significantly different from normal curve expectations at either time points.

We found a higher percentage of “at risk” status for girls with TS on a variety of social communication functions at 12 months of age, particularly in the areas of symbolic and social language. Specifically, a high percentage of our infants were found to be “at risk” on the CSBS-DP Behavior Sample for the functions of social communication, social gestures, and symbolic object use. This was not the case for the 24-month-old girls with TS whose “at risk” percentages were nonsignificant when compared to normative data. While findings on the CSBS-DP Caregiver Questionnaire at the 12-month time point were similar to those on the CSBS-DP Behavior Sample regarding symbolic use of objects, two other behaviors, social emotion and eye gaze, and symbolic understanding, emerged as having a high percentage of girls “at risk.” This was not the case for the 24-month-old girls with TS; the percentages of those “at risk” were found to be nonsignificant when compared to normative data. Our findings do not provide clear reasons for the mastery of these early skills; however, it is possible that they improved due to early stimulation or intervention and/or that temperamental differences at 12 months of age [35] improved and no longer interfered with the acquisition of social and symbolic language. An alternative explanation is that by the time girls with TS are 24 months of age, they have already mastered early social and symbolic communication skills and have progressed developmentally into linguistic communication where there are different social pragmatic language skills to learn.

### Changes in language abilities from 12 to 24 months of age

Finally, when examining the changes in core language abilities from 12 to 24 months, using a subset of our 12-month-old infants with TS who had a follow-up evaluation at 24 months, we found that, although there was a slight downward trend in core language scores from 12 to 24 months, receptive, expressive, and total language scores remained relatively stable. This also was true for those who were in the “at risk” category. There was a positive shift in social language abilities (i.e., CSBS-DP) on all composite and scaled scores from 12 to 24 months of age. There were significant improvements noted over the 1-year period in speech composite, speech words, social emotion and eye gaze, and symbolic use of objects based on the behavior sample as well as in social gestures and speech sounds based on the Caregiver Questionnaire. There also were changes that included both increases and decreases in the percentage of girls who were “at risk” for social language difficulties, but the changes in the “at risk” classification were overall not significant.

### Discrepancies between CSBS-DP and PLS-4 total scores and total “at risk” percentages

After examining the results of our analyses, we questioned why there might be differences between the CSBS-DP and PLS-4 total scores and total “at risk” percentages. We surmise that the differences in total language and total standard scores between measures and “at risk” percentages may be due, in part, to inconsistent infant/toddler performance and test variability. In addition, while the PLS-4 measures core language skills, the CSBS-DP focuses on measurement of speech, social, and symbolic communication. The CSBS-DP surveys communication skills but also often-overlooked indicators of symbolic development, including gestures, facial expressions, and play behaviors. While the PLS-4 includes some gestures and some play on both AC and EC subtests, other indicators, such as eye gaze, facial expression, social bids for behavior regulation, social interaction, and joint attention, are minimally included in the PLS-4. This perhaps accounts for some of the apparent differences between the PLS-4 and CSBS-DP test results (i.e., the measures are assessing different aspects of language and communication across respondents).

### Limitations

First and foremost, the results of this study should be interpreted with caution due to the small sample size and it is likely that our TS cohort may not be a random sample of the TS population at large. Second, participants were recruited from across the USA, and participation was dependent on a family’s ability and willingness to travel, thus leading to a sample of convenience and potential bias. Third, this study did not have a comparison group, and, therefore, we capitalized on the strong normative data for each measure. A matched group of typically developing peers would have afforded a one-to-one comparison of the findings across each of the time points and, perhaps, more precision in the findings. In that regard, an ideal comparison group would include typically developing infants and toddlers or females with other conditions, but that type of sample is difficult to obtain. In addition to adding comparison groups, future work should aim to control for factors such as race/ethnicity, maternal depression, medical factors/procedures (e.g., heart surgery), and height/weight.

## Conclusions

This study represents early efforts in understanding the language and communication skills of very young children with TS. Although variable across time points, results suggest that receptive language, in particular, as well as social communication and symbolic communication areas, should be considered part of routine developmental follow-up with children with TS. Evaluation of a child for a communication disorder should focus not only on core language abilities, but also on social and symbolic abilities, both verbal and nonverbal [18], as they may represent the first indicators of later language difficulty. These indicators also may be difficult to identify without direct assessment and ongoing developmental surveillance. Hopefully, these preliminary findings will lay the foundation for future intensive study of the language development of infant and toddler girls with TS, and even more specifically, the social pragmatic language functions expected of 24-month-old girls with TS, particularly with respect to the connectedness of these early functions with communication and language abilities during the preschool, school-age, and adolescent years.

## Data Availability

The datasets generated and/or analyzed during the current study are not publicly available due to lack of funding to prepare the data set for public access but are available via direct contact with the PI, Rebecca Knickmeyer on reasonable request.

## Abbreviations

TS: Turner syndrome
SRS: Social Responsiveness Scale
TD: Typically developing
Mullen: Mullen Scales of Early Learning
PLS-4: Preschool Language Scale, Fourth Edition
CSBS-DP: Communication and Symbolic Behavior Scales, Developmental Profile
SES: Socioeconomic status
DQ: Developmental quotient
SD: Standard deviation
FDR: False-discovery rate
AC: Auditory comprehension
EC: Expressive communication
ASD: Autism spectrum disorder
